# Electro-Optical Comb Envelope Engineering Based on Mode Crossing

**DOI:** 10.3390/ma17051190

**Published:** 2024-03-04

**Authors:** Shuting Kang, Xiaomin Lv, Chen Yang, Rui Ma, Feng Gao, Xuanyi Yu, Fang Bo, Guoquan Zhang, Jingjun Xu

**Affiliations:** 1 The MOE Key Laboratory of Weak Light Nonlinear Photonics, School of Physics and TEDA Applied Physics Institute, Nankai University, Tianjin 300457, China; 1120210094@mail.nankai.edu.cn (S.K.); zhanggq@nankai.edu.cn (G.Z.); jjxu@nankai.edu.cn (J.X.); 2 State Key Laboratory for Artificial Microstructure and Mesoscopic Physics and Frontiers Science Center for Nano-Optoelectronics, School of Physics, Peking University, Beijing 100871, China

**Keywords:** lithium niobate, electro-optic comb, integrated photonics

## Abstract

Resonator-enhanced electro-optical (EO) combs could generate a series of comb lines with high coherence and stability. Recently, EO comb based on thin-film lithium niobate (TFLN) has begun to show great potential thanks to the high second-order nonlinearity coefficient of lithium niobate crystal. Here we demonstrate that EO comb envelope engineering based on mode crossing induced a quality factor reduction in the TFLN racetrack microcavity both in the numerical simulation and experiment. Our method paves the way for the generation of EO combs with an arbitrary envelope.

## 1. Introduction

The optical frequency comb (OFC) acts as a crucial bridge between optical and microwave domains, offering a precise method for frequency measurement. Traditional microcavity OFCs are often based on third-order nonlinearities in material platforms and are thus called Kerr combs. They exploit degenerate and non-degenerate four-wave mixing (FWM) to generate cascaded comb lines. Achieving phase-locked soliton states involves balancing dispersion and nonlinearity, as well as gain and loss [1,2]. Kerr combs have been developed in various material platforms, such as Si_3_N_4_ [3], MgF_2_ [4], AlN [5], Silicon [6], LiNbO_3_ [7], and more. They find wide applications in areas such as range measurement [8,9], lidar [10], optical clocks [11], optical communication [12], and quantum optics [13]. Soliton microcombs possess the advantages of a small size and compatibility with wafer-scale processing. However, Kerr combs pose integration challenges due to their demand for high on-chip power and precise pump frequency tuning for stable soliton states. Addressing these challenges would facilitate their broader integration into various applications.

In contrast to Kerr combs, the electro-optic (EO) comb utilizes the second-order nonlinear effects of materials [14]. It generates cascaded sidebands by phase modulation in waveguides or microcavities, resulting in periodic comb lines around the pump [15,16]. EO comb’s properties can be altered by adjusting modulation frequency, amplitude, or the number of cascaded modulators. EO comb can be briefly divided into two categories: resonant devices and non-resonant devices. As for resonant devices, electrodes are placed along the straight section of a high-quality microcavity, and the modulation frequency matches the FSR of the resonator for the generation of a wide-range comb. The resonant-enhanced EO comb offers lower power consumption and improved integration. Unlike resonant EO combs relying on high-quality resonators, non-resonant EO combs are generally more flexible and exhibit a flat spectral response [17,18]. They can arbitrarily adjust the pump wavelength and RF frequency over a large range without the limitation of FSR. On the other hand, they often have relatively low bandwidth and fewer comb lines in relation to resonant EO combs. Unlike Kerr combs, resonant-enhanced EO combs demand less on-chip power while maintaining commendable coherence and stability. This makes them a promising advancement in EO comb technology, particularly beneficial for applications in communication systems where reduced power consumption and improved integration are highly desirable. The adaptability of EO combs through pump frequency adds a layer of flexibility, catering to diverse application requirements.

Thin-film lithium niobate (TFLN), as one of the primary platforms in integrated optics, has gained favor among researchers due to its outstanding properties. The lithium niobate has a large transparency window (400 nm–5 μm), strong second-order nonlinearity (*d*_33_ = 34 pm/V), the ability of periodic poling, and a high-speed optical switch through the EO effect [19]. Many high-performance optical devices based on TFLN have been demonstrated, such as low-pump wavelength convertors [20,21,22], tunable frequency combs [23,24,25], and EO modulators with a high frequency and low driving voltage [26,27].

EO comb is widely applied on the X-cut TFLN platform due to the high EO coefficient [16,17,28]. High-performance EO combs based on TFLN have been applied to dual-comb spectroscopy [29] and frequency mirrors [30], which show great potential in integrated optics. In particular, the utilization of EO combs in dual-comb spectroscopy exemplifies their capability to provide precise and rapid measurements of optical spectra. Additionally, the implementation of EO combs in frequency mirrors demonstrates their adaptability for signal processing and manipulation, further extending their utility in integrated optics applications [30]. The widespread adoption of EO combs on the X-cut TFLN platform signifies the platform’s prominence in developing advanced optical devices. The combination of TFLN’s favorable material properties and the unique capabilities of EO combs holds promise for continued advancements in integrated optics, opening possibilities for novel applications and breakthroughs in optical communication, sensing, and signal processing.

The envelope of OFCs is crucial in practical applications, especially in the field of communication. Typically, only a few comb lines are needed for communication, and the other frequency components are filtered out using band-pass filters. This not only increases energy consumption, leading to the wastage of a portion of pump power, but also adds complexity to the optical path with the inclusion of off-chip filtering components. Unlike Kerr solitons, which exhibit an almost unchangeable sech^2^function envelope [31], the spectral envelope of an EO comb could be engineered by adding an auxiliary cavity or using mode crossing [30]. Until now, there has not been a clear solution or theoretical simulation results for engineering the envelope of an EO comb. K. Zhang et al. achieved mode splitting at the design wavelength of a racetrack resonator by designing a photonic crystal cavity [32], enabling a frequency mirror at a specific wavelength. This approach provides insight into designing the envelope of an EO comb by controlling the resonance frequency of a mode at a specific wavelength. The method involves careful design of the resonator shape for controlling mode splitting in photonic crystal microcavities, with higher requirements for fabrication techniques.

Here, we propose a method for OFC envelope engineering using mode crossing within a microcavity. In certain situations, it is desirable to avoid mode crossing because we aim for single-mode transmission in the microcavity, and mode crossing will cause the spectral envelope of solitons to become non-smooth, which is unfavorable for communication and other applications [31,33]. However, in some cases, mode crossing can be advantageous. For instance, it can be utilized for the generation of solitons in ring cavities and the low-threshold generation of optical parametric oscillators (OPOs), which can be beneficial in experimental setups [34,35,36]. Mode crossing does not impose strict fabrication requirements on the microcavity, and the distinct effective refractive indices along different directions in the X-cut TFLN microcavity make mode crossing more achievable. This provides a method to build an arbitrary envelope of EO comb. Previously, mode crossing in X-cut TFLN was employed in the design of mode converters, with limited research being conducted on intracavity mode conversion. However, intracavity mode conversion is a crucial issue. On other material platforms, designing Euler curves [37] or modified Euler curves [38] has proven effective in reducing intracavity mode coupling.

In this article, we explore the mode-crossing-induced manipulation of the EO comb envelope based on TFLN. We theoretically analyzed the envelope changes caused by local loss, conducted numerical simulations, and fabricated racetrack cavity samples using X-cut lithium niobate. The locally added extra loss is realized by mode crossing in the microcavity in the experiment. The test results demonstrated good agreement between the experimental envelope and the theoretical predictions.

## 2. Theory and Numerical Simulation

**Figure 1:**
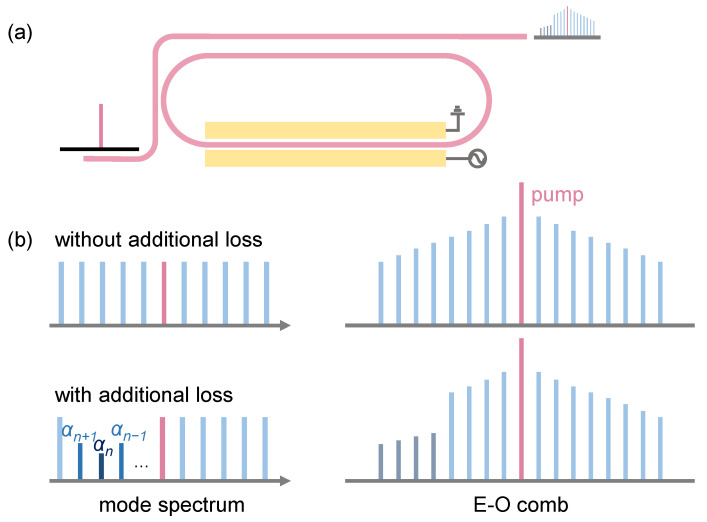
(**a**) Schematic diagram of an EO comb generated through RF modulation. (**b**) Schematic diagrams of the smooth EO comb corresponding to cavity modes with consistent loss coefficients α, and the EO comb with dips around mode number *n* caused by cavity modes with additional losses around the mode number *n*.

The schematic diagram of the generation of the EO comb can be observed in Figure 1a. By applying a continuous wave (CW) EO phase modulation to the microcavity, an EO comb can be generated around the pump light with a fixed space FSR. The modulation frequency should be equal to the FSR of the cavity to generate resonant-enhanced comb lines. We depict in Figure 1b that in consideration of envelope engineering, a mode-crossing-induced loss could be added to the comb lines with mode number *n*, and *n* ± 1, thus producing the cavity mode with loss $\gamma_{n}$, $\gamma_{n+1}$, and $\gamma_{n-1}$, respectively. Under the influence of losses, the envelope of the EO comb lines will exhibit a dip at *p* = *n*, causing the comb teeth to truncate at the desired wavelength, where *p* is the index of the comb lines.

In consideration of the pump input with

(1) $$E_{in}\left(t\right)=\tilde{E_{in}}e^{i\omega_{0}t}$$

where $\omega_{0}$ is the resonant frequency of the racetrack microcavity. Then, if each mode is on resonance, the output could be written as [39]

(2) $$E_{out}\left(t\right)=\sqrt{\left(1-\gamma \right)\left(1-k\right)}E_{in}\left(t\right)-k\sqrt{\frac{1-\gamma }{1-k}}\frac{re^{i\beta \sin\omega_{m}t}}{1-re^{i\beta \sin\omega_{m}t}}E_{in}\left(t\right)$$

where γ is the power insertion loss, and *k* is the power transmission. The cavity is modulated with the radio frequency (RF) of $\omega_{m}$ and modulation index β. $r=\sqrt{\alpha (1-\gamma )(1-k)}\;$is the round-trip gain. α represents the remaining energy of the optical field after completing one round inside the cavity, and thus 1 − α indicates the loss experienced during one round. If each comb line experiences the same energy loss α, the α in Equation (2) will be simplified to a constant. However, if there is an additional cavity mode loss $\alpha_{n}$ at p=n, then *α* will become an *p* × 1 matrix with $\alpha =(\alpha_{1},\alpha_{2},...\alpha_{n-1},\alpha_{n},\alpha_{n+1},...\alpha_{p}{)}^{T}$. The additional loss could be added by mode crossing in the application. When two sets of modes are simultaneously coupled into the cavity, the degenerate resonant frequency could lead to the shift of resonant wavelengths or mode splitting, thereby causing variations in the microcavity dispersion and a decrease in the quality factor at a certain wavelength.

By adding the matrix of *α* to the steady-state electric field in the frequency domain, we could obtain the relation of all the comb lines

(3) $$E_{p}=r_{p}\sum_{q=-\infty }^{\infty }J_{q}\left(\beta \right)E_{p-q}e^{i\theta_{p-q}}+i\sqrt{\frac{k}{1-k}}r_{p}\tilde{E_{in}}J_{p}\left(\beta \right)e^{i\theta_{0}}$$

where $\theta_{p}=\theta_{p,0}+\theta_{p,m}+\theta_{p,d\;}$ is the round-trip phase offset. $\theta_{p,0}$ is the original phase, and $\theta_{p,m}$, $\theta_{p,d}$ is the phase offset caused by RF modulation and dispersion of the *p^th^* comb lines, respectively. *E_p_* could be solved quickly using a convolution method. It is worth mentioning that convolution should be performed with a matrix larger than the number of combs to ensure the accuracy of the result.

**Figure 2:**
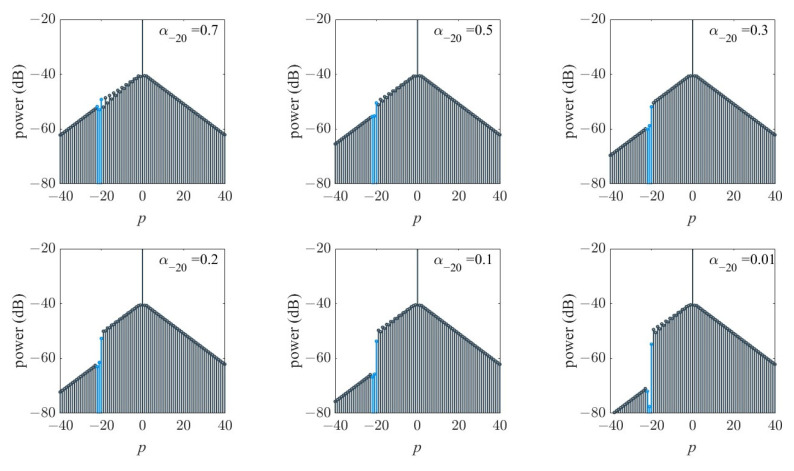
Loss-induced envelope dip from $\alpha_{n}=0.7$ to $\alpha_{n}=0.01$, where *n* = −20 and $\alpha_{n\pm 1}=\alpha_{n}+0.1$. The colored lines depict the loss position according to comb line number *p*, where *k =* 0.03, *γ =* 0.

We first add the loss to the 20th comb lines and take no account of dispersion. In consideration of generating a frequency reflector within a single microcavity, the loss caused by mode crossing should occur not only at a specific resonance wavelength but at several modes near the overlap. The farther the distance from the overlap center, the smaller the loss. Here, we assume the loss around the center loss line *p_n_* with $\alpha_{n\pm 1}=\alpha_{n}+0.1$, and the numerical simulation results can be seen in Figure 2. A clear dip appeared at the loss mode, and the dip depth increased when the alpha decreased from 0.7 to 0.01.

**Figure 3:**
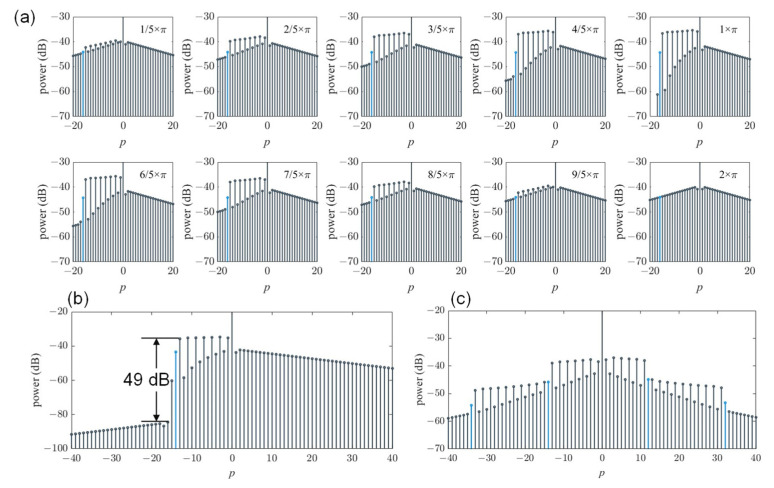
(**a**) Comb spectrum with the phase detuning from π/5 to 2π with an interval of π/5. The lines that are marked represent the comb lines with an added phase term $\phi_{ext}$. (**b**) An enlarged view of the graph in Figure (**a**) when $\phi_{ext}\;$*= π* shows the 49 dB extinction ratio. (**c**) The constructed frequency mirror achieved by adding four phase terms $\phi_{ext}=\pi /2$ to the comb line.

We also added a phase term $\phi_{ext}$ to a single comb line to generate a frequency mirror. Figure 3a shows the comb spectrum with the phase detuning from $\frac{\pi }{5}$ to 2π. The comb spectrum for $\phi_{ext}=0$ is the same as $\phi_{ext}=2\pi$, which is easy to comprehend because the period of the exponential function is 2π according to Equation (3). When $\phi_{ext}=\pi$, the envelope drops off apparently, and the detailed spectrum envelope can be seen in Figure 3b with a 49 dB extinction ratio. This phenomenon can be regarded as a frequency mirror with tunable reflectivity. Then, we added multiple phase terms and generated a frequency mirror at different wavelengths with different reflectivity, shown in Figure 3c.

It is noteworthy that in this article, we did not include the dispersion term in the simulations. This is because in practical testing, the envelope of the comb teeth has already dropped to the noise floor of the optical spectrum analyzer (OSA) before being influenced by dispersion, given the relatively low-quality factors of the resonant cavity. In subsequent fabrication processes, it will be essential to explore parameters for constructing resonant cavities with higher-quality factors to reduce losses.

## 3. Experiment Results

We fabricated the racetrack resonator with electrodes based on a 600 nm X-cut TFLN. The etch depth was 350 nm for better dispersion of the EO comb. The waveguide and cavity width were 1 μm and 1.4 μm, respectively. The pattern was defined using electron beam lithography (EBL) with hydrogen silsesquioxane (HSQ) as the resist and etched using Inductively Coupled Plasma Reactive Ion Etching (ICP-RIE) with Ar ion milling. Then, the device was cleaned with buffered HF solution and RCA1 cleaning solution to remove the remaining HSQ resist and the redeposition formed in the etching process. A 200 nm Au electrode was deposited on TFLN based on electronic beam evaporation without another buffer layer. The gap between the signal and ground electrodes is 5 μm. A larger gap tends to reduce the propagation losses of the waveguide, but it concurrently decreases the RF modulation efficiency. Choosing an appropriate gap is crucial in the design process of EO combs to balance these competing factors effectively. It is worth noting that we think the electrode spacing chosen here is somewhat small, contributing to the lower Q of the racetrack resonator. In future experiments, we will make improvements in this regard and explore an appropriate modulation gap.

**Figure 4:**
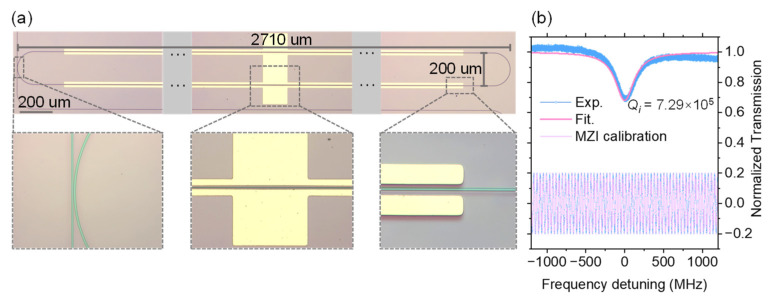
(**a**) Waveguide and electrode details of the fabricated racetrack microcavity with a footprint of 200 × 2710 μm^2^. The radius of the curved section is 100 μm. (**b**) Zoomed-in view of the resonances with Lorentzian fits indicating *Q_i_* of 7.29 × 10^5^. The interference curve of MZI is displayed below, which is utilized for calibrating the laser’s sweep speed and exhibits a periodic sinusoidal shape.

The racetrack resonator was designed with a footprint of 2710 × 200 μm, which corresponds to an FSR of around 25 GHz to match the amplification center of the RF amplifier. The details of the cavity and electrodes are shown in Figure 4a. Quality factor (*Q*) was measured using an arbitrary function generator at a center wavelength of 1549.88 nm. A 5 m long Mach–Zehnder Interferometer (MZI) was used for calibrating the wavelength scanning rate to obtain an accurate *Q*. The FSR of MZI is around 40.78 MHz, which is enough for frequency calibration and cavity linewidth measurement. Figure 4b shows the cavity with intrinsic *Q* (*Q_i_*) of 7.29 × 10^5^ and the details of the MZI calibration curve.

**Figure 5:**
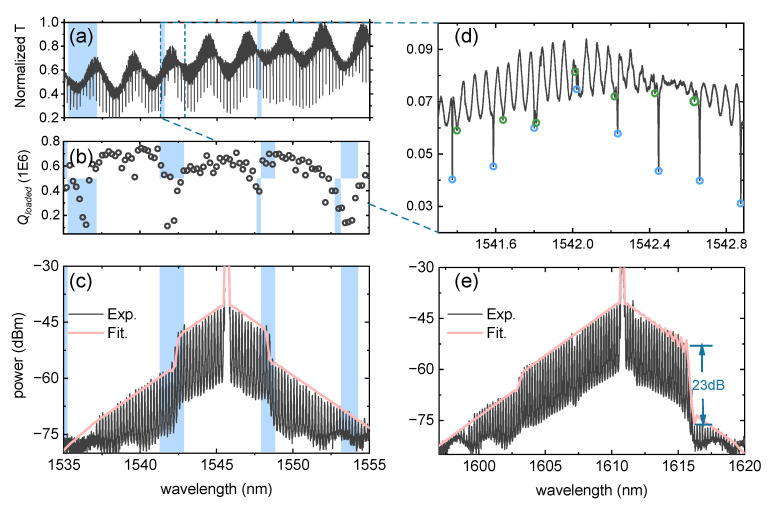
(**a**) Transmission spectrum of racetrack cavity from 1535 nm to 1555 nm. The blue-shaded region depicts the mode crossing region. (**b**) The counted *Q_loaded_* of each resonance peak. The shaded region shows the mode-crossing-induced *Q* decrease. (**c**) The experiment measured EO comb lines and fitted envelope with the same extinction ratio at both sides of the pump wavelength. (**d**) A magnification of (**a**) of the mode crossing region. The blue and green circle depicts two sorts of modes. (**e**) The experimental result and numerical fitting curve of EO comb with distinct extinction ratio at different sides of the pump wavelength.

To verify our numerical simulation results, we measured the wide spectrum of the racetrack cavity and the clipped wide spectrum from 1535 nm to 1555 nm, as shown in Figure 5a. The *Q_loaded_* was then analyzed by Lorentz curve fitting. The input wavelength of the cavity was then fixed at 1545.691 nm with an on-chip power of about 2 mW. RF signal was applied to the cavity using an RF signal generator and amplified by an RF amplifier. We measured the EO comb spectrum using an optical spectrum analyzer (OSA), and the comb with a frequency span width of 20 nm can be seen in Figure 5c.

The horizontal axis wavelengths of Figure 5a–c are consistent. We can observe that when mode crossing occurs, there is a decrease in *Q*, and at this point, a dip appears in the spectral envelope of the EO comb. The decrease in the quality factor at mode crossing is not limited to a specific wavelength; instead, several resonant peaks at that wavelength experience a certain degree of *Q* reduction. This aligns with the assumptions made in our numerical simulations. In Figure 5d, we plotted the wide spectrum near the dip on the left side of the electro-optic comb and marked the two groups of overlapping modes with blue and green circles. When the degree of overlap between the two groups of modes is large, the linewidth of the transmission peak increases, and the *Q* value decreases. This is indeed the main reason for the sudden decrease in the spectral envelope of the electro-optic comb, consistent with our theoretical analysis. We set the loss of comb lines at both sides of the pump wavelength t$o\;\alpha_{i}$ = 0.3 and $\alpha_{i\pm 1\;}$ = 0.4. The theoretical fit of the envelope closely matches the experimental results, providing evidence for the reliability of our theory.

Additionally, we employed our theoretical model to fit an EO comb with disparate dip depths on both sides in experiments with another cavity in Figure 5e. The cavity has the same parameters of the cavity and electro with the cavity in Figure 4a but a different cavity–waveguide gap. The fitting results align well with the experimental data. The fitting parameters on the left and right sides are $\alpha_{left}$ = 0.6 and $\alpha_{right}$ = 0.1, respectively. The extinction ratio at the mode crossing point reached 23 dB, showing great performance of our method.

In our experiments, we observed periodic mode overlapping, causing the FSR to exhibit a periodic fluctuation in the region of mode crossing. We selected wide spectrum data ranging from 1596 nm to 1610 nm of another cavity for clarity. It can be observed that in the presence of mode crossing, the FSR shows fluctuations of approximately 0.4 GHz, attributed to the resonance wavelength shift caused by mode crossing. The period of the fluctuation at around 1603 nm is approximately 4.5 nm. We think that this fluctuation is due to coherent interference between the fundamental mode and other modes within the cavity. Considering the optical path difference between the two modes inside the cavity, we can derive

(4) $$\Delta f=\frac{\Delta \omega }{\Delta \phi }=\frac{c}{\Delta s\left(n_{g1}-n_{g0}\right)}$$

where *n_g_*_1_ and *n_g_*_0_ are the group refractive indices of the two sets of modes involved in the interaction. Δ*s* is the path length, which we set to 2710 nm in consideration of the horizontal section of the cavity footprint.

**Figure 6:**
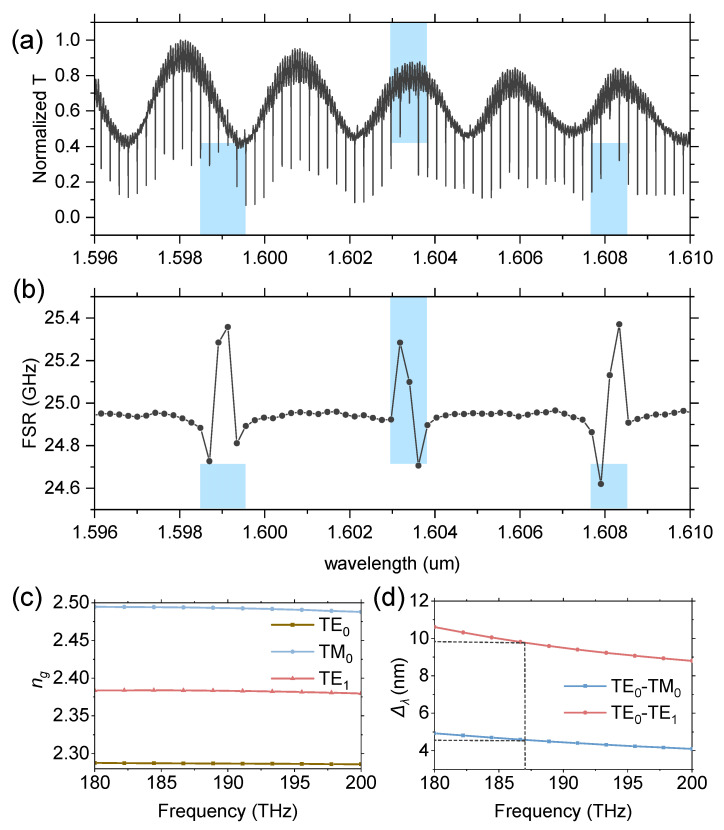
(**a**) Transmission spectrum of a racetrack cavity from 1596 nm to 1610 nm. The blue-shaded region depicts the mode crossing region. (**b**) The FSR changes with wavelength. Blue-shaded region indicates the mode-crossing-induced FSR fluctuation. (**c**) Calculated *n_g_* for TE_0_, TM_0_, and TE_1_ modes. (**d**) Calculated Δ*_λ_* for TE_0_-TM_0_ modes and TE_0-_TE_1_ modes. The crosspoint marks the wavelength of 1603 nm with Δ*_λ_* of 9.76 nm and 4.57 nm, respectively.

Utilizing the Finite Difference Eigenmode (FDE) method, we calculated the *n_g_* for different wavelengths of TE_0_, TM_0_, and TE_1_ modes in Figure 6c. The waveguide width was set to 1.4 μm, which is equal to the cavity width in the experiment. The interference period can be calculated using Equation (4). In Figure 6d, we present the interference periods between the TE_0_ mode and TM_0_ mode, as well as between the TE_1_ and TE_0_ modes. At the wavelength of 1603 nm, the interference period between TE_0_ and TM_0_ is 4.53 nm, matching the fluctuation period of the FSR at 4.5 nm. Meanwhile, the interference period between TE_0_ and TE_1_ is 9.76 nm. Therefore, we attribute the two modes causing mode crossing to the TE_0_ and TM_0_ modes. The TM_0_ mode inside the resonator is converted from the TE_0_ mode through the curved section of the cavity. Reducing mode crossing or achieving more precise control can be accomplished by designing the curvature shape of the cavity. 

We note here that the mode crossing period in Figure 5 is approximately 5 nm, which is slightly larger than our calculated period of 4.5 μm. We attribute this discrepancy to fabrication errors. Additionally, the simplification of the curved section of the cavity into a straight waveguide during the period calculation is another source of inaccuracy of the calculated result.

## 4. Conclusions

In conclusion, we theoretically analyzed the mode spectrum envelope changes caused by the decrease in the *Q* of certain modes in the racetrack cavity. We experimentally validated these findings, and there is excellent agreement between the experimental and theoretical results. The racetrack resonator used here has high modulation efficiency because of the long racetrack section. On other material platforms such as silicon for EO combs, ring cavities are preferred, and similarly, our theoretical model could be used for envelope engineering through dispersion design. Moreover, an asymmetric microcavity could realize a broadband momentum transformation assisted by chaotic motions [40], which could be applied to the disk cavity or the bulk lithium niobate crystal cavity for generating a broadband EO comb.

Our theory is applicable to envelopes with losses at arbitrary wavelengths and intensities, making it suitable for the inverse engineering of the mode spectra. The engineering range of a single racetrack cavity is limited. To extend this capability, we could explore the design of photonic crystal microcavities or incorporate an auxiliary cavity for coupling control in the future, enabling spectrum engineering at the specified wavelengths. Or explore the design of ring width and shape to achieve better control over mode crossing. Our method provides opportunities to enhance the performance of communication devices. The intentional manipulation of mode crossing in TFLN microcavities opens up possibilities for advanced applications in optical communication and signal processing. Additionally, by optimizing the design parameters, such as ring width and shape, we can further tailor the behavior of mode crossing, enabling the realization of novel and high-performance devices for various photonic applications.

Up to now, material platforms such as InP [41], SOI [42], and SiN [43] could also generate the EO comb. Our comb shaping method could also be applied to these material platforms if the EO comb is generated by the micro-ring cavity. In addition, lithium tantalate could also be a good platform for EO comb generation and envelope engineering due to its giant Pockels coefficient (*r*_33_ = 30.5 pm/V) [44].

## Data Availability

The data presented in this study are available on request from the corresponding author.
